# Openness, neuroticism, conscientiousness, and family health and aging concerns interact in the prediction of health-related Internet searches in a representative U.S. sample

**DOI:** 10.3389/fpsyg.2014.00370

**Published:** 2014-04-29

**Authors:** Tim Bogg, Phuong T. Vo

**Affiliations:** Department of Psychology, Wayne State UniversityDetroit, MI, USA

**Keywords:** personality, Big Five, openness, stress, aging, health information, Internet search

## Abstract

Recent estimates suggest 60% of the U.S. adult population uses the Internet to find health-related information. The goal of the present study was to model health-related Internet searches as a function of an interdependent system of personality adaptation in the context of recent health and aging-related concerns. Assessments of background factors, Big Five personality traits, past-month health and aging-related concerns, and the frequency of past-month health-related Internet searches (via Google, Yahoo, AOL, Bing, or some other search engine) were obtained from a representative U.S. sample (*N =* 1,015). Controlling for background factors, regression analyses showed more frequent health-related Internet searches were predicted by a drive for exploration and investigation (high openness), as well as alarm sensitivity (high openness and high neuroticism) and an anticipatory inclination (high openness and high conscientiousness) in the context of recent problems with aging parents and recent health concerns for a family member. Consistent with interdependent models of personality adaptation, as well as prior evidence for “surrogate” health-related Internet searches, the results suggest a personality process model of search behavior that is partially dependent upon dispositional levels of exploration, emotional stability, control, and health and aging concerns for family members.

## INTRODUCTION

According to a recent report by the Pew Internet Project, 80% of U.S. adult Internet users have searched for health information online (Fox, 2011). Excluding the approximately 25% of the at-large adult population that does not use the Internet, nearly 60% of adults have conducted online health information searches. Background factors, such as greater educational attainment, income, and age (those older than 65 years are less likely to search) are known to be associated with health-related Internet search rates (Weaver et al., 2009; Fox, 2011; Miller and Bell, 2011). Recent research also suggests that, in addition to seeking information related to one’s own health-related concerns, searches for information related to close others’ health and well-being – surrogate searching – is a common form of Internet-based health-information-seeking behavior (Sadasivam et al., 2013). While these findings suggest some of the demographic and structural factors contributing to searches, the person-centered characteristics – such as personality traits and current life concerns – that might also contribute to health and aging-related searches are largely unknown.

An interdependent system of personality adaptation – one that emphasizes interactions among major personality traits in response to internal and external inputs, such as stressors or feedback (Van Egeren, 2009; Weiss et al., 2009; DeYoung, 2010) – offers a framework for examining how traits might be associated with this emergent, yet highly prevalent, health-related behavior. Moreover, re-casting current life concerns as specific health and aging-related stressors related to the self and close others might provide a clearer account of the internal and external circumstances by which varying levels of personality traits contribute to the use of searches as a form of personality adaptation. The goal of the present study is to examine an interdependent system of personality adaptation for recent health-related Internet searches.

## THE HEALTH RELEVANCE OF AN INTERDEPENDENT SYSTEM OF PERSONALITY ADAPTATION

In recent years, interdependent models of major personality trait domains (i.e., the Big Five; Goldberg, 1993) have received increasing attention (Hirsh et al., 2009; Van Egeren, 2009; Weiss et al., 2009; DeYoung, 2010). These models borrow, in part, from cybernetic feedback control theory (Weiner, 1948), which describes how machines exert control over their functioning in response to inputs in order to meet self-regulatory goals and purposes (e.g., an oven maintaining a set temperature; autopilot reliably controlling the flight of a commercial airliner). As it relates to a system of personality adaptation, these models designate independent *and* interdependent functioning of trait domains to facilitate actions that enable goal pursuit. The emphasis on interdependent effects is consistent with recent research on personality styles, where combinations or profiles of trait domains have been found to be uniquely associated with health-related outcomes (e.g., Weiss et al., 2009; Turiano et al., 2013). From a cybernetic perspective, the purpose of adaptive responses in the personality system – whether independent or interdependent – is to foster the pursuit of valued goals (e.g., maintaining one’s physical health) that might otherwise be thwarted if no corresponding action is produced.

Using the lens of interdependence, differing levels of traits, as well as differing levels of different combinations of traits, can be expected to correspond to varying levels of the same responses or even widely divergent responses. For example, if the demanding maintenance for Type 2 Diabetes is the health-related input, then concomitant internal (e.g., increased rumination versus calm resignation) and external (e.g., missing timely blood glucose checks versus closely monitoring one’s diet) responses could be expected to vary in their presence and intensity based on individual differences in neuroticism (worried versus stable) and conscientiousness (responsible versus lazy). It should be noted that there is no universal benchmark for adaptation. A response for an individual with low neuroticism and high conscientiousness might be composed diligence. In contrast, a response for an individual with high neuroticism and low conscientiousness might be anxious inaction. The functionality of the response is a separate consideration from the form of the response itself and requires an understanding of the context in which the response was produced.

## HEALTH-RELATED INTERNET SEARCHES AS AN EMERGENT PERSONALITY ADAPTATION STRATEGY FOR HEALTH AND AGING CONCERNS

Although widely prevalent and shown to be associated with age, education, and other background/demographic factors, health-related Internet searches are poorly understood from a psychological perspective. Framing such searches as a partial function of an interdependent system of personality adaptation allows for an examination of *a priori* expectations regarding the contributions of trait domains and internal and external inputs/stressors to the discrete, yet complex behavior of using an Internet search engine to query for health- and aging-related information. The modeling of these relations necessitates a consideration of the combinations of traits and internal and external health and aging concerns that might correspond to health-related search behavior. The expected relations can be separated in two ways: (1) the independent contributions of trait domains to search behavior, irrespective of specific internal and external health-related inputs and (2) the interdependent contributions of trait domains to search behavior in the context of specific internal and external health-related inputs.

### INDEPENDENT CONTRIBUTIONS OF EACH BIG FIVE TRAIT TO SEARCH BEHAVIOR

In the absence of intervening internal or external contexts to a system of personality adaptation, the relation of a single trait domain to an response strategy is posited to be a function of the dispositional drive of that domain – that is, the comparatively non-specific deployment of responses to satisfy endogenous levels of drive for a specific trait.

One of the primary regulatory functions ascribed to extraversion is enabling reward- and approach-related actions and behaviors (Van Egeren, 2009; DeYoung, 2010). Without a clear incentive or arousing features, it seems unlikely that individual differences in extraversion would be associated with health-related Internet searches. Similarly, without an apparent context for cooperation, empathy, or closeness, it also seems unlikely that individual differences in agreeableness would be associated with health-related Internet searches. Moreover, in the absence of defined tasks, competing environmental demands, or temporal forbearance, it also seems unlikely individual differences in conscientiousness would be associated with search behaviors.

In contrast, one of the primary regulatory functions ascribed to neuroticism is error information signaling and processing (Carver and Scheier, 1990; Lahey, 2009; Van Egeren, 2009). By definition, heightened levels of this general drive should be sufficient to prompt adaptive action and behavior in the absence of evidence of setbacks, threats, or outright failure, especially given that neurotic individuals are more likely to perceive themselves to be afflicted in some way, even when they are healthy (Watson and Pennebaker, 1989). Individual differences in the general propensity to view the world and oneself as faulty, error-prone, or dissatisfying seem likely to be associated with health-related Internet search behaviors.

The primary regulatory functions ascribed to openness are encountering, exploring, and parsing various forms of abstract and sensory information (Van Egeren, 2009; DeYoung, 2010; DeYoung et al., 2012). Heightened levels of the drive to extract, retrieve, and utilize information should be sufficient to prompt adaptive action and behavior, even in the absence of internal or external factors that might otherwise cue investigative tendencies. It is expected that openness should be positively associated with health-related Internet searches – a platform ready-made for such a dispositional drive.

### INTERDEPENDENT CONTRIBUTIONS OF BIG FIVE TRAITS AND HEALTH AND AGING CONCERNS TO SEARCH BEHAVIOR

Given the expectations of independent contributions of neuroticism and openness to search behaviors, these two trait domains become viable candidates for inclusion in the larger interdependent model of personality adaptation that corresponds to search behavior – a model that provides a rendering of interactions among the domains in response to internal and external inputs or stressors. Based on factor-analytic research with the Big Five, as well as recent postulating, two meta-traits – stability and plasticity – are posited to be superordinate mechanisms that monitor, control, and adapt system functioning so that goals can be pursued and achieved (Digman, 1997; Hirsh et al., 2009; DeYoung, 2010). Stability is represented by aspects of neuroticism, conscientiousness, and agreeableness; it provides a mechanism whereby error and goal control, monitoring, and detection can be achieved. Plasticity is represented by aspects of extraversion and openness; it provides a mechanism whereby responses to internal and external dynamic circumstances can be facilitated. In the current study, the internal and external contexts are recent experiences of physical pain, physical illness, health issues of a family member, and problems with aging parents.

Consistent with the stability and plasticity meta-traits, the presence of contexts for the system’s goals, such as recent health and aging concerns, would be perceived as threatening or aversive by individuals high in neuroticism, whose dispositional drive produces a greater sensitivity to potential instability. For individuals high in neuroticism who are also experiencing a recent health and aging concern (i.e., presence of input), an increase of the adaptive response of health-related Internet searches would be contingent upon levels of openness, where high levels of openness should be associated with the greatest frequency of search behavior. Conversely, individuals high in neuroticism and openness who are not experiencing a recent health and aging concern would not experience the same level of response amplification, although it would still be expected to be greater than that of individuals low in neuroticism and openness who are also experiencing a recent health and aging concern. To summarize, a three-way interaction among neuroticism, openness, and recent health and aging concerns is expected, such that the most searches will be conducted by individuals with recent health and aging concerns scoring high in neuroticism and openness.

Although no independent effect of conscientiousness on search behavior is expected, given the error- and goal-control drives ascribed to the domain (Van Egeren, 2009; DeYoung, 2010), it seems likely that individuals with recent health and aging concerns scoring high in conscientiousness and openness would see an increase in search behavior compared with individuals low in conscientiousness and openness. In the context of recent health and aging concerns, the combination of high conscientiousness (active goal control monitoring) and high openness (active information exploration) in the presence of a recent health and aging concern might correspond to greater search behavior as part of a more systematic response, where search behavior is one of several potential pathways to goal control and dynamic accommodation of evolving circumstances. Whereas high openness has a naturally affinitive (and independent) link with search behavior, the link between high conscientiousness and search behavior is expected to be conditional. The expected three-way interaction among conscientiousness, openness, and health and aging concerns represents a process whereby recent health and aging concerns are filtered by the conscientiousness-related drives for error and goal control to prompt an adaptive response, which, in the context of high openness, should be associated with the most frequent recent search behavior.

## MATERIALS AND METHODS

### PARTICIPANTS

The survey was conducted using the web-enabled Knowledge Panel^®^, a probability-based national panel designed to be representative of the U.S. population. Initially, participants were chosen by a random selection of telephone numbers and residential addresses. Persons in selected households were then invited by telephone or by mail to participate. For those who agreed to participate, but did not already have Internet access, GfK Custom Research LLC provided a laptop and ISP connection at no cost. People who already had computers and Internet service were permitted to participate using their own equipment. Panelists then received unique log-in information for accessing surveys online (completion rate: 63.4%). GfK maintains an agreement with each panelist ensuring the voluntary completion of each survey (and each survey item), as well as the option to decline to respond to survey items, decline to participate in a given survey, and/or be entirely removed from the panel without penalty. In the current study, participants (*N* = 1,015) ranged from 18–88 years of age, with a mean age of 46.51 years (*SD* = 17.12 years). The sample was sex-balanced (51.8% females) and the majority of the participants were White, Non-Hispanic (66.7%).

### ASSESSMENT MATERIALS

#### Education, income, and household Internet access

Highest level of education attained was assessed using scores representative of one of 14 categories (1 = No formal education, 14 = Professional or Doctorate degree). Income was assessed using scores representative of one of 19 categories (1 = Less than $5,000; 19 = $175,000 or more). Household Internet access was assessed using a dichotomous variable indicating the presence or absence of Internet access in the residence.

#### Self-rated health, health-related limitations

Self-rated health was assessed with a single item using a five-point Likert scale (In general, would you say your health is? 1 = Poor, 5 = Excellent). Ten items from the SF-36 (Ware and Sherbourne, 1992) were used to assess limitations in daily physical activities resulting from health problems (e.g., lifting or carrying groceries, climbing stairs, and bending, kneeling, or stooping; α = 0.95).

#### Recent self and other health and aging concerns

Items from Kanner et al.’s (1981) hassles scale were used to assess the presence or absence of health and aging concerns during the past-month (i.e., three single items assessing health of a family member, physical illness, and problems with aging parents). Hassles were defined as “irritants that can range from minor annoyances to fairly major pressures, problems, or difficulties.” One item from the SF-36 was used to measure participants’ experience of pain in the past-month (“During the past four weeks, how much bodily pain have you experienced?”). Participants rated pain on a six-point Likert scale (1 = None, 6 = Very severe).

#### Big Five Inventory (BFI)

The well-validated 44-item BFI was used to assess five broad domains of personality traits (John et al., 1991). All items were rated using a five-point Likert scale (1 = Disagree Strongly, 5 = Agree Strongly). An eight-item scale was used to assess neuroticism (e.g., “gets nervous easily”; α = 0.85). A 10-item scale was used to assess openness (e.g., “is curious about many different things”; α = 0.80). A nine-item scale was used to assess extraversion (e.g., “is outgoing, sociable”; α = 0.81). A nine-item scale was used to assess conscientiousness (e.g., “does a thorough job”; α = 0.81). An eight-item scale was used to assess agreeableness (e.g., “is helpful and unselfish with others”; α = 0.81).

#### Recent health-related Internet searches

A single item was used to assess the frequency of health-related internet searches in the past-month using a six-point scale (i.e., “In the past-month, how often have you used Google, Yahoo, Bing, AOL (or some other search engine) to search the Internet for health-related information?” 0 = Never, 1 = Once in past-month, 2 = Weekly, 3 = A few times per week, 4 = Daily, 5 = More than once per day).

### ANALYTIC APPROACH

Sampling weights were used in all analyses to modify the sample characteristics to be representative of the U.S. population. This weighting procedure adjusted for survey non-response, as well as non-coverage, under- or over-sampling, or participant demographic factors (sex, age, race/ethnicity, education, census region, household income, residence in a metropolitan area, and Internet access).

Correlational analyses were used to examine the strength and direction of associations among the study variables. It should be noted that the frequency of health-related Internet searches variable showed evidence of a skewed distribution. To address this, raw scores were subjected to a Blom transformation, which rank orders the raw scores (settling ties by using the mean of the contested ranks) and then transforming the ranks to z scores using the normal distribution. Simulation research analyzing several transformation types showed that a Blom transformation of symptom count data (i.e., skewed data similar the health-related Internet search data in the present study) resulted in a more accurate selection of a true model from a set of alternative models (van den Oord et al., 2000).

Initial regression analyses were conducted using three sets of approaches: linear regression with raw search scores, linear regression with Blom-transformed search scores, and ordinal regression with raw search scores. The three sets of approaches yielded highly similar results, consistent with evidence for the robustness of linear regression in the presence of deviations from normality, especially in larger data sets (e.g., *N* > 500; *cf.* Lumley et al., 2002). As a result, for purposes of clear presentation and interpretability, the results for the linear regression with raw score data are presented here.

Two sets of regression models were constructed and analyzed to examine the independent and interdependent effects of the Big Five traits and recent health and aging concerns on health-related Internet searches. The first set of models simultaneously examined the associations of (1) background (including sex and minority status), self-rated health, and limitations variables, (2) recent health and aging concerns, (3) Big Five personality traits, and (4) the interactions among neuroticism, openness, and recent health and aging concerns to recent health-related Internet searches. The second set of models included the first three components of the first set of models, but differed in its examination of the interactions among conscientiousness, openness, and recent health and aging concerns in the prediction of recent health-related Internet searches. To summarize, two sets of four separate models were examined, one for each set of two-way and three-way interaction terms among the relevant Big Five traits and the recent health and aging concerns. Consistent with the guidelines for testing interaction effects by Jaccard and Turrisi (2003), all variables were standardized (i.e., *z*-scored) prior to entry into the models.

## RESULTS

### DESCRIPTIVE ANALYSES

Means (or percentages) and standard deviations for the study variables appear in **Table 1**. On average, the sample reported experiencing very mild amounts of pain in the past-month. Approximately one quarter of the sample reported experiencing a physical illness in the past-month. A similar percentage reported experiencing an issue related to the health of a family member. Approximately one seventh of the sample reported experiencing problems with aging parents in the past-month. On average, participants reported searching the Internet for health-related information slightly less than one time per week in the past-month. The modal response was no searches in the past-month (29.4%), followed by once in the past-month (21.6%), more than once per week in the past-month (15.4%), once per week in the past-month (13.5%), daily during the past-month (11.3%), and more than once per day in the past-month (8.8%).

**Table 1:** Descriptive statistics for study variables.

Study variables (response ranges)	Mean (SD) or Percentage
Age (18–88)	46.51 (17.12)
Income (1–19)	11.67 (4.59)
Education (1–14)	10.08 (2.10)
In-home Internet (yes/no)	75%
Self-rated health (1–5)	3.33 (0.92)
Physical limitations (0–20)	3.77 (5.30)
Past-month pain (1–6)	2.74 (1.33)
Past-month illness (yes/no)	23%
Past-month family member health (yes/no)	29%
Past-month aging parent problems (yes/no)	14%
Extraversion (1–5)	3.09 (0.75)
Neuroticism (1–5)	2.76 (0.81)
Conscientiousness (1–5)	3.84 (0.65)
Agreeableness (1–5)	3.83 (0.67)
Openness (1–5)	3.36 (0.62)
Past-month Internet health searches (0–5)	1.84 (1.66)

### CORRELATIONAL ANALYSES

**Table 2** displays the correlations among the study variables. Consistent with prior research, younger individuals, those with greater income and education, and those with in-home Internet access tended to conduct more frequent past-month health-related Internet searches. Greater self-rated health was associated with greater frequency of past-month health-related searches. Past-month experiences of pain and physical illness were not associated with past-month health-related searches. In contrast, past-month experiences of concerns regarding the health of a family member and problems with aging parents were associated with more frequent past-month health-related searches. Greater openness was associated with more frequent past-month health-related searches. However, the association between neuroticism and past-month searches was not significant at the bivariate level.

**Table 2:** Correlations among study variables.

	1	2	3	4	5	6	7	8	9	10	11	12	13	14	15
1. Age	-														
2. Income	-0.05	-													
3. Education	-0.02	0.35^*^	-												
4. In-home Internet	-0.15^*^	0.45^*^	0.25^*^	-											
5. Self-rated health	-0.12^*^	0.24^*^	0.20^*^	0.12^*^	-										
6. Physical limitations	0.21^*^	-0.27^*^	-0.20^*^	-0.20	-0.43^*^	-									
7. Past-month pain	0.16^*^	-0.13^*^	-0.19^*^	-0.08^*^	-0.45^*^	0.51^*^	-								
8. Past-month illness	0.06	-0.12^*^	-0.02	-0.01	-0.36^*^	0.31^*^	0.43^*^	-							
9. Past-month family member health	0.13^*^	-0.07^†^	0.06	0.06	-0.10^*^	0.04	0.04	0.16^*^	-						
10. Past-month aging parent problems	0.02	0.05	0.09^*^	0.04	-0.02	0.03	0.04	0.12^*^	0.30^*^	-					
11. Extraversion	0.03	0.09^*^	-0.02	0.03	0.25^*^	-0.09^*^	-0.09^*^	-11^*^	-0.09^*^	-0.05	-				
12. Neuroticism	-0.12^*^	-0.09^*^	-0.13^*^	-0.06	-0.32^*^	0.13^*^	0.22^*^	0.22^*^	0.08^*^	0.08^*^	-0.27^*^	-			
13. Conscientiousness	0.20^*^	0.13^*^	0.13^*^	0.06	0.20^*^	-0.15^*^	-0.04	-0.04	0.06	0.07^†^	0.21^*^	-0.38^*^	-		
14. Agreeableness	0.17^*^	0.06^†^	0.11^*^	0.04	0.13^*^	-0.13^*^	-0.05	0.01	0.06^†^	0.04	0.15^*^	-0.40^*^	0.48^*^	-	
15. Openness	0.00	-0.02	0.12^*^	0.08^*^	0.23^*^	-0.14^*^	-0.08^*^	-0.03	0.02	0.05	0.32^*^	-0.15^*^	0.24^*^	0.15^*^	-
16. Past-month Internet health searches	-0.13^*^	0.09^*^	0.12^*^	0.13^*^	0.06^†^	0.02	0.01	0.00	0.12^*^	0.14^*^	0.05	0.05	0.02	0.02	0.22^*^

*Note.*

* p < 0.01, †p < 0.05.

### REGRESSION MODELS

Consistent with expectations and the correlational results, and controlling for background factors, recent health concerns for a family member, recent problems with aging parents, and greater neuroticism and openness were associated with more frequent health-related Internet searches (see **Table 3**). Recent pain was not predictive of the frequency of health-related Internet searches in the regression models.

**Table 3:** Additive and interactive effects of openness, neuroticism, conscientiousness, problems with aging parents, and health concerns for a family member on health-related Internet searches.

	Past-month Internet health searches		
	Model 1 (R = 0.383, *r*^2^ = 0.147)	Model 2 (R = 0.383, *r*^2^ = 0.147)	Model 3 (R = 0.379, *r*^2^ = 0.144)
	B	B	B
Sex	-0.03	0.00	0.00
White vs. Non-White	-0.14^*^	-0.14^*^	-0.16^*^
Age	-0.24^*^	-0.24^*^	-0.23^*^
Income	0.10	0.12	0.11
Education	0.17^*^	0.16^*^	0.18^*^
In-home Internet	0.10	0.09	0.09
Self-rated health	0.03	0.03	0.04
Limitations	0.21^*^	0.22^*^	0.22^*^
Past-month pain	0.07	0.06	0.06
Past-month physical illness	-0.13^*^	-0.13^*^	-0.13^*^
Past-month family member health	0.17^*^	0.19^*^	0.21^*^
Past-month aging parent problems	0.15^*^	0.18^*^	0.13^*^
Extraversion	-0.02	-0.02	-0.02
Neuroticism	0.19^*^	0.15^*^	0.16^*^
Conscientiousness	0.00	0.00	0.00
Agreeableness	0.07	0.08	0.08
Openness	0.34^*^	0.37^*^	0.36^*^

**Model 1**	**B**	**Model 2**	**B**	**Model 3**	**B**
N × O	-0.13^*^	C × O	0.05	C × O	0.06
N × AgePar	-0.01	C × AgePar	-0.06	C × FamHealth	0.06
O × AgePar	0.00	O × AgePar	0.05	O × FamHealth	-0.04
N × O × AgePar	0.11^*^	C × O × AgePar	-0.15^*^	C × O × FamHealth	-0.10^*^

* p < 0.05. Regression models were constructed using simultaneous entry of all terms. Bs are interpretable in outcome units (frequency of searches). O = Openness, N = Neuroticism, C = Conscientiousness.

#### Neuroticism, openness, and recent health and aging concerns

**FIGURE 1:**
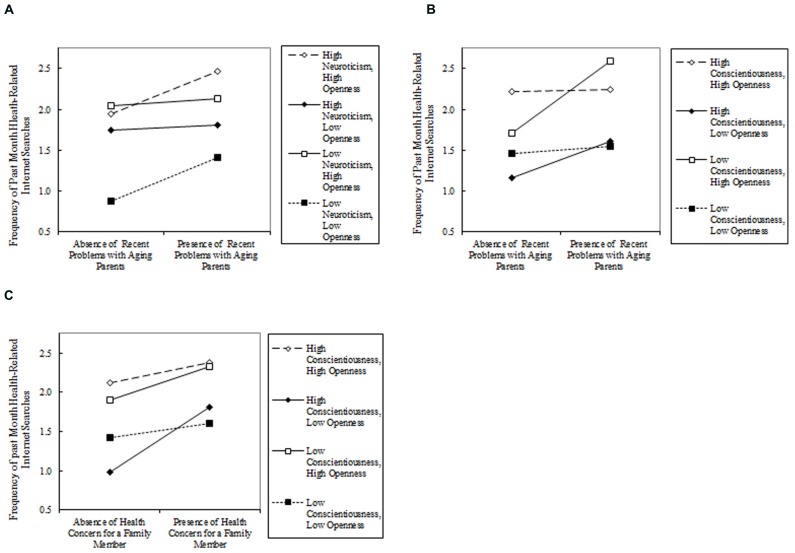
**(A)** depicts the three-way interaction among high and low ( +/- 1 standard deviation) levels of openness, neuroticism, and the absence/presence of past-month problems with aging parents in the prediction of past-month health-related Internet searches. **(B)** depicts the three-way interaction among high and low ( +/- 1 standard deviation) levels of openness, conscientiousness, and the absence/presence of past-month problems with aging parents in the prediction of past-month health-related Internet searches. **(C)** depicts the three-way interaction among high and low ( +/- 1 standard deviation) levels of openness, conscientiousness, and the absence/presence of past-month health concerns for a family member in the prediction of past-month health-related Internet searches.

A three-way interaction was identified among neuroticism, openness, and recent problems with an aging parent (see **Figure 1A**). The form of the interaction for neuroticism, openness, and recent problems with an aging parent suggests the combination of high levels of neuroticism and openness, in combination with the presence of recent problems with an aging parent, was associated with a greater frequency of recent health-related Internet searches. In other words, anxious and curious individuals who experienced problems with aging parents tended to conduct more health-related Internet searches than similarly disposed individuals who had not reported experiencing problems with aging parents. The least frequent amount of recent health-related Internet searches was conducted by individuals scoring low on neuroticism and openness who reported no recent problems with aging parents.

#### Conscientiousness, openness, and recent health and aging concerns

A three-way interaction was identified among conscientiousness, openness, and recent problems with an aging parent (see **Figure 1B**). A three-way interaction also was identified among conscientiousness, openness, and recent health concerns for a family member (see **Figure 1C**). The form of these interactions suggests the combination of high levels of conscientiousness and openness were associated with comparatively greater search frequency, regardless of the presence of problems with an aging parent or health concerns for a family member. Individuals low in conscientiousness and high in openness showed the largest increase in search frequency in the presence of recent problems with aging parents compared to similarly disposed individuals who had not experienced recent problems with aging parents. Individuals high in conscientiousness and low in openness showed the largest increase in search frequency in the presence of recent health concerns for a family member compared to similarly disposed individuals who had not experienced recent health concerns for a family member.

## DISCUSSION

The goal of the present study was to examine an interdependent system of personality adaptation for health-related Internet searches, controlling for known and novel background factors and incorporating recent health and aging concerns to better understand the contextual concomitants of search behavior. Consistent with prior research, background factors such as age, income, education, and household Internet access were associated with the frequency of past-month searches (Weaver et al., 2009; Fox, 2011; Miller and Bell, 2011). Moreover, search behavior was associated with recent concerns and problems related to the health and well-being of family members and parents. These findings provide clarifying context for prior research that identified surrogate searching as a major form of querying, but could not address the life circumstances associated with such searches (Sadasivam et al., 2013). For the personality attributes examined, the Big Five trait domain of openness showed the strongest independent prediction of search behaviors of any of the study variables. In addition, interactions among openness, neuroticism, conscientiousness, problems with aging parents, and health concerns for a family member provided evidence for interdependent effects of dispositional drives and external inputs on regulatory responses in a system of adaptation. The implications for these findings, as well as the strengths and limitations of the current study are discussed below.

### INVESTIGATIVE AND EXPLORATORY DRIVES PREDICT HEALTH-RELATED INTERNET SEARCHES

To date (and to our knowledge), only one other study examined associations between the Big Five trait domains and health-related Internet searches. In a sample of adults in their sixties from Wisconsin, openness was found to be associated with having ever used the Internet to search for health information (Flynn et al., 2006). As the authors noted, the findings from the sample were not easily generalizable and the timeframe for search behavior was imprecise. The finding for openness in the current study using a representative U.S. sample, which specified a search timeframe, lends credence to this earlier finding.

The moderately strong independent effect for openness also speaks to the robustness of the dispositional drive to pursue and explore information that is ascribed to the openness domain, as well as its role in the plasticity mechanism of interdependent models of personality adaptation (Barrick et al., 2003; Van Egeren, 2009; DeYoung et al., 2012). That is, even in the absence of contextual inputs that might cue or prompt search behavior, individuals high in openness are more apt to be querying for health- and aging-related information. This appears to be one of the few realms of health-related behaviors or outcomes where the trait domain of openness plays a significant role, especially as compared to neuroticism and conscientiousness (*cf.* Ozer and Benet-Martínez, 2006; Lahey, 2009; Bogg and Roberts, 2013). This finding also suggests a novel pathway in health-related personality research – one that affords a unique role to openness in the acquisition of health- and aging-related information. While openness appeared to play a predominant role in health-related searches, the results also showed its effects likely need to be understood through its interactions with neuroticism, conscientiousness, recent problems with aging parents, and health concerns for a family member.

### ALARM SENSITIVITY AND ANTICIPATORY INCLINATION CHARACTERIZE INTERDEPENDENT TRAIT EFFECTS FOR INCREASED HEALTH-RELATED INTERNET SEARCHES

Consistent with the regulatory role of neuroticism as an alerting mechanism for system instability, the results showed individuals high in neuroticism and openness conducted the most searches in the presence of recent problems with aging parents, as compared to similarly disposed individuals who were not experiencing problems with aging parents. In the absence of a drive to alert for instability and a dearth of exploratory drive (i.e., low neuroticism and low openness, respectively), the contextual strength of problems with aging parents corresponded to a modest increase in search behavior, although the comparative level of searching was still lower than all other trait-context combinations.

Although there was increased frequency of searching at high levels of openness and conscientiousness, there was no concomitant increase in search behavior in the presence of problems with aging parents or health concerns for a family member. These patterns suggest individuals with high levels of goal control/monitoring in combination with high levels of exploration/investigation do not show a discriminatory response in the presence of problems with aging parents or health concerns for a family member, at least in terms of increased search behavior. It may be the case that, unlike the combination with high levels of neuroticism and openness, high levels of conscientiousness and openness represent a chronically high level of adaptive response – an anticipatory inclination – that shows little adjustment to the presence of recent problems with aging parents or health concerns for a family member. A similar pattern emerged for the combination of low conscientiousness and low openness. At very low levels of anticipatory readiness (i.e., low goal control/monitoring, low exploratory/investigative drive), there is a chronically low level of adaptive response that remains relatively static in the presence of problems with aging parents or health concerns for a family member, at least in terms of search behavior.

### LIMITATIONS AND IMPLICATIONS

The use of a representative U.S. sample – assessed on a comprehensive set of background variables, health and aging concerns, and personality traits – marks a substantive advancement in depicting health-related Internet searches. Moreover, the framing of health-related Internet searches as part of a interdependent system of personality adaptation represents a novel application of a personological perspective for such behavior. In spite of these strengths, the study is not without limitations.

Aside from the sole reliance on self-reported measures, the primary weakness of the present work is the uncertain replicability of the complex three-way interaction effects. In spite of the robustness of these effects across different regression approaches and transformations of the outcome variable, interaction effects, in general – and especially non-experimental three-way interaction effects – can be illusory. As a result, the patterns depicted in **Figure 1** should be interpreted as preliminary and requiring replication under highly similar sampling and measurement conditions.

Although the Big Five were measured using a reliable and valid assessment instrument, the scales are, by design, intended to provide broad coverage of each domain. It may be the case that narrower facets of openness (e.g., ingenuity, Goldberg, 1999; ideas; Costa and McCrae, 1992), neuroticism (e.g., anxiety, Costa and McCrae, 1992; stability; Goldberg, 1999), and conscientiousness (organization, purposefulness, Goldberg, 1999; deliberation, self-discipline, Costa and McCrae, 1992) would provide increased predictive and conceptual utility. In addition, given the relevance of medical mistrust to a variety of health outcomes, a narrower facet of agreeableness, such as (low) trust (Costa and McCrae, 1992), might demonstrate an association with search behavior – an association informed by skepticism and suspicion for other sources of information, such as healthcare providers.

Although the health and aging concerns assessed in the current study pertained to both internal (e.g., pain) and external (e.g., problems with aging parents) sources, they were not exhaustive. An assessment of coping strategies would likely aid in understanding the interdependent effects (*cf.* Carver and Connor-Smith, 2010). For example, endorsement of emotion-focused coping strategies might mediate the moderated association between neuroticism and search behavior. Likewise, endorsement of problem-focused coping strategies might mediate the moderated relationship between conscientiousness and search behavior.

Finally, health-related Internet searches represent just one possible adaptive response to the presence of health and aging concerns. A consideration of a range of possible responses, including consulting with a physician, attempting self-remedies, or seeking advice from close others, would provide a more thorough depiction of a model of personality adaptation in the context of health and aging concerns.

As is suggested by the results, alarm sensitivity (high openness-neuroticism) seems to be responsive to eldercare concerns. Caregivers of aging parents or other elderly family members often experience a variety of physical and psychosocial burdens (Pinquart and Sörensen, 2006). Individuals high in openness and neuroticism may find that pursuing information on the Internet is a means of both targeted exploration of eldercare issues, as well as coping with the concern via resource gathering. By contrast, anticipatory inclination (high openness and conscientiousness) contributes to a high steady state of health-related searches that is not responsive to health and aging concerns. Individuals with this combination of attributes appear to conduct searches to satisfy drives for exploration and goal control, where searching might produce patterns of information that could be used for the immediate gratification of curiosity and the efficiency of future reference.

Consistent with the larger goal of understanding how personality predicts consequential outcomes (i.e., how it “gets outside the skin”; Hampson, 2012), the results of the current study suggest the importance of utilizing personality process frameworks for health-related behaviors, especially those that emphasize personality styles and interdependent relations among major trait domains (e.g., Van Egeren, 2009; Weiss et al., 2009; Turiano et al., 2013). More concretely, the findings suggest the important role of the openness domain – exploratory versus close-minded – in health- and aging-related Internet search behavior, and possibly health information acquisition more generally. This finding suggests the importance of openness to health literacy – an important marker of health outcomes linked to increased hospitalizations, increased healthcare spending, lower use of preventive care, and poorer health status (Baker et al., 2002; Nielsen-Bohlman et al., 2004; Howard et al., 2005).

Far from being solely a function of education, income, or access, the results of the current work point to the importance of dispositional attributes – especially openness – and recent contextual factors in predicting the common, but complex behavior of querying a search engine for health-related information.

## Conflict of Interest Statement

The authors declare that the research was conducted in the absence of any commercial or financial relationships that could be construed as a potential conflict of interest.

## References

[B1] Baker D. W. Gazmararian J. A. Williams M. V. Scott T. Parker R. M. Green D. (2002). Functional health literacy and the risk of hospital admission among Medicare managed care enrollees. *Am. J. Public Health* 92 1278–1283 10.2105/AJPH.92.8.1278 12144984 PMC1447230

[B2] Barrick M. R. Mount M. K. Gupta R. (2003). Meta-analysis of the relationship between the Five-Factor model of personality and Holland’s occupational types. *Pers. Psychol.* 56 45–74 10.1111/j.1744-6570.2003.tb00143.x

[B3] Bogg T. Roberts B. W. (2013). The case for conscientiousness: evidence and implications for a personality trait marker of health and longevity. *Ann. Behav. Med*. 45 278–288 10.1007/s12160-012-9454-6 23225322 PMC3604184

[B4] Carver C. S. Connor-Smith J. (2010). Personality and coping. *Annu. Rev. Psychol.* 61 679–704 10.1146/annurev.psych.093008.100352 19572784

[B5] Carver C. Scheier M. (1990). Origins and functions of positive and negative affect: a control process view. *Psychol. Rev.* 97 19–35 10.1037//0033-295X.97.1.19

[B6] Costa P. T. Jr. McCrae R. R. (1992). NEO *PI-R Professional Manual*. Odessa, FL: Psychological Assessment Resources

[B7] DeYoung C. G. (2010). Toward a theory of the Big Five. *Psychol. Inq.* 21 26–33 10.1080/10478401003648674

[B8] DeYoung C. G. Grazioplene R. G. Peterson J. B. (2012). From madness to genius: the Openness/Intellect trait domain as a paradoxical simplex. *J. Res. Pers.* 46 63–78 10.1016/j.jrp.2011.12.003

[B9] Digman J. M. (1997). Higher-order factors of the Big Five. *J. Pers. Soc. Psychol.* 73 1246–1256 10.1037/0022-3514.73.6.1246 9418278

[B10] Flynn K. E. Smith M. A. Freese J. (2006). When do older adults turn to the internet for health information? Findings from the Wisconsin Longitudinal Study. *J. Gen. Intern. Med*. 21 1295–1301 10.1111/j.1525-1497.2006.00622.x 16995892 PMC1924748

[B11] Fox S. (2011). *Health Topics. Pew Internet and American Life Project.* Available at: http://pewinternet.org/Reports/2011/HealthTopics.aspx (accessed September 20, 2013)

[B12] Goldberg L. R. (1993). The structure of phenotypic personality traits. *Am. Psychol.* 48 26–34 10.1037//0003-066X.48.1.26 8427480

[B13] Goldberg L. R. (1999). “A broad-bandwidth, public domain, personality inventory measuring the lower-level facets of several five-factor models,” in *Personality Psychology in Europe* eds Mervielde I. Deary I. De Fruyt F. Ostendorf F. (Tilburg, The Netherlands: Tilburg University Press) 7–28

[B14] Hampson S. E. (2012). Personality processes: mechanisms by which personality traits “get outside the skin”. *Annu. Rev. Psychol.* 63 315–339 10.1146/annurev-psych-120710-100419 21740225 PMC3193854

[B15] Hirsh J. B. DeYoung C. G. Peterson J. B. (2009). Metatraits of the Big Five differentially predict engagement and restraint of behavior. *J. Pers.* 77 1085–1102 10.1111/j.1467-6494.2009.00575.x 19558442

[B16] Howard D. H. Gazmararian J. Parker R. M. (2005). The impact of low health literacy on the medical costs of Medicare managed care enrollees. *Am. J. Med*. 118 371–377 10.1016/j.amjmed.2005.01.010 15808134

[B17] Jaccard J. Turrisi R. (2003). *Interaction Effects in Multiple Regression,* 2nd Edn, (Sage University Papers Series on Quantitative Applications in the Social Sciences, 07-072). Thousand Oaks, CA: Sage

[B18] John O. P. Donahue E. M. Kentle R. L. (1991). *The Big Five Inventory–Versions 4a and 54*. Berkeley, CA: University of California Press

[B19] Kanner A. D. Coyne J. C. Schaefer C. Lazarus R. S. (1981). Comparison of two modes of stress management: daily hassles and uplifts versus major life events. *J. Behav. Med.* 4 1–39 10.1007/BF00844845 7288876

[B20] Lahey B. B. (2009). Public health significance of neuroticism. *Am. Psychol.* 64 241–256 10.1037/a0015309 19449983 PMC2792076

[B21] Lumley T. Diehr P. Emerson S. Chen L. (2002). The importance of the normality assumption in large public health data sets. *Annu. Rev. Public Health* 23 151–169 10.1146/annurev.publheath.23.100901.140546 11910059

[B22] Miller L. M. S. Bell R. A. (2011). Online health information seeking: the influence of age, information trustworthiness, and search challenges. *J. Aging Health* 24 525–541 10.1177/0898264311428167 22187092

[B23] Nielsen-Bohlman L. Panzer A. M. Kindig (2004). *Health literacy: A prescription to end confusion*. Washington, DC.: The National Academies Press. 25009856

[B24] Ozer D. J. Benet-Martínez V. (2006). Personality and the prediction of consequential outcomes. *Annu. Rev. Psychol.* 57 401–421 10.1146/annurev.psych.57.102904.190127 16318601

[B25] Pinquart M. Sörensen S. (2006). Gender differences in caregiver stressors, social resources, and health: an updated meta-analysis. *J. Gerontol. B Psychol. Sci. Soc. Sci.* 61B 33–45 10.1093/geronb/61.1.P33 16399940

[B26] Sadasivam R. S. Kinney R. L. Lemon S. C. Shimada S. L. Allison J. J. Houston T. K. (2013). Internet health information seeking is a team sport: analysis of the Pew Internet Survey. *Int. J. Med. Inform*. 82 193–200 10.1016/j.ijmedinf.2012.09.008 23149121

[B27] Turiano N. A. Mroczek D. K. Moynihan J. Chapman B. P. (2013). Big 5 personality traits and interleukin-6: evidence for “healthy Neuroticism” in a U.S. population sample. *Brain Beh. Immun.* 29 83–89 10.1016/j.bbr.2011.03.031 PMC3545072 23123863

[B28] van den Oord E. J. C. G. Simonoff E. Eaves L. J. Pickles A. Silberg J. Maes H. (2000). An evaluation of different approaches for behavior genetic analyses with psychiatric symptom scores. *Beh. Genetics.* 30 1–18 10.1023/A:1002095608946 10934795

[B29] Van Egeren L. F. (2009). A cybernetic model of global personality traits. *Pers. Soc. Psychol. Rev.* 13 92–108 10.1177/1088868309334860 19351887

[B30] Ware J. E. Jr. Sherbourne C. D. (1992). The MOS 36-item short-form health survey (SF-36): I. Conceptual framework and item selection. *Med. Care* 30 473–483 10.1097/00005650-199206000-00002 1593914

[B31] Watson D. Pennebaker J. (1989). Health complaints, stress, and distress: exploring the role of negative affectivity. *Psychol. Rev.* 96 234–254 10.1037//0033-295X.96.2.234 2710874

[B32] Weaver J. B. III Mays D. Lindner G. Eroglu D. Fridinger F. Bernhardt J. M. (2009). Profiling characteristics of internet medical information users. *J. Am. Med. Inform. Assoc*. 16 714–722 10.1197/jamia.M3150 19567794 PMC2744722

[B33] Weiner N. (1948). *Cybernetics of Control and Communication in the Animal and the Machine*. New York: John Wiley

[B34] Weiss A. Sutin A. R. Duberstein P. R. Friedman B. Bagby R. M. Costa P. T. Jr. (2009). The personality domains and styles of the five-factor model are related to incident depression in medicare recipients aged 65 to 100. *Am. J. Geriatr. Psychiatry.* 17 591–601 10.1097/JGP.0b013e31819d859d 19554673 PMC2745829

